# Prognostic DNA Methylation Markers for Prostate Cancer

**DOI:** 10.3390/ijms150916544

**Published:** 2014-09-18

**Authors:** Siri H. Strand, Torben F. Orntoft, Karina D. Sorensen

**Affiliations:** Department of Molecular Medicine, Aarhus University Hospital, Brendstrupgaardsvej 100, 8200 Aarhus N, Denmark; E-Mails: Siri.Strand@clin.au.dk (S.H.S.); Orntoft@clin.au.dk (T.F.O.)

**Keywords:** prostate cancer, DNA methylation, epigenetics, biomarker, prognosis

## Abstract

Prostate cancer (PC) is the most commonly diagnosed neoplasm and the third most common cause of cancer-related death amongst men in the Western world. PC is a clinically highly heterogeneous disease, and distinction between aggressive and indolent disease is a major challenge for the management of PC. Currently, no biomarkers or prognostic tools are able to accurately predict tumor progression at the time of diagnosis. Thus, improved biomarkers for PC prognosis are urgently needed. This review focuses on the prognostic potential of DNA methylation biomarkers for PC. Epigenetic changes are hallmarks of PC and associated with malignant initiation as well as tumor progression. Moreover, DNA methylation is the most frequently studied epigenetic alteration in PC, and the prognostic potential of DNA methylation markers for PC has been demonstrated in multiple studies. The most promising methylation marker candidates identified so far include *PITX2*, *C1orf114* (*CCDC181*) and the *GABRE~miR-452~miR-224* locus, in addition to the three-gene signature *AOX1/C1orf114/HAPLN3*. Several other biomarker candidates have also been investigated, but with less stringent clinical validation and/or conflicting evidence regarding their possible prognostic value available at this time. Here, we review the current evidence for the prognostic potential of DNA methylation markers in PC.

## 1. Introduction

Prostate cancer (PC) is a common malignancy and a major health care problem in Western countries, where up to one in six men will be diagnosed with PC during their lifetime [1,2]. While most PC patients have a slowly progressing tumor with little or no clinical manifestation, other patients suffer from aggressive PC characterized by metastatic dissemination and almost invariably lethal outcome [3]. As a consequence of the widespread use of PSA (prostate specific antigen) testing, most PCs are currently diagnosed at an early organ-confined stage, when curative treatment is still possible by e.g., radical prostatectomy (RP). Nevertheless, more than 30% of RP patients suffer biochemical recurrence (BCR) within 10 years of surgery, many of whom will later develop metastatic castration-resistant PC (CRPC) that is highly morbid and incurable [4]. On the other hand, exaggerated use of PSA testing has led to overdiagnosis and overtreatment of many clinically insignificant PCs, reducing the quality of life for many men due to treatment-associated side effects such as impotence and incontinence, as well as unnecessary anxiety [5].

Distinction between aggressive and indolent disease remains a major challenge for the clinical management of PC, as there are no biomarkers or prognostic tools that can accurately predict tumor progression at the time of diagnosis. Currently available nomograms for PC prognosis are based almost exclusively on routine clinicopathological parameters, *i.e.*, serum PSA, clinical stage, and Gleason score [6,7]. Furthermore, although several nomograms exist, there is no accepted gold standard and many newly diagnosed PC patients are misclassified, leading to suboptimal treatment decisions [8]. Moreover, available clinical nomograms generally do not include molecular events, despite growing evidence for the prognostic value of such molecular biomarkers [9].

Indeed, several promising prognostic molecular tests are beginning to emerge for PC. These include commercial tests based on gene expression measurements, such as the “Prolaris” test that stratifies risk of PC progression based on a 46-gene expression signature [10], and the “MiProstate Score” that estimates the likelihood of aggressive PC by combining urinary measurements of PCA3 and TMPRSS2-ERG transcripts with serum PSA levels [11]. However, none of these assays are as of yet FDA-approved but are available under a Clinical Laboratory Improved Amendments (CLIA) certificate [12]. DNA methylation markers have also shown very promising prognostic potential for PC [13,14,15,16], and thus could be excellent candidates for inclusion into improved prognostic nomograms, either alone or in combination with other types of molecular markers. In this review, we will focus specifically on the prognostic potential of aberrant DNA methylation in relation to PC.

Epigenetic modifications are defined as heritable and reversible biochemical changes affecting gene expression without altering the primary DNA sequence. The most intensively studied epigenetic modification is DNA methylation, which is nearly ubiquitous in multicellular organisms and essential for normal development in mammals [17]. Methylation of the 5' carbon of cytosine (5mC) in CpG dinucleotides in gene promoters is regarded as the most direct epigenetic mechanism for maintaining gene silencing [18]. While CpG dinucleotides are underrepresented at the genomic scale, clusters of CpG dinucleotides (termed CpG islands (CGIs)) are found in the promoter region of approximately half of the genes in the human genome [19]. In normal cells, promoter-associated CGIs are generally unmethylated—a state that is permissive but not sufficient for active gene expression. Consistent with this, DNA methylation is believed to primarily be a mechanisms for long-term gene silencing seen mainly at imprinted, X-inactivated, and germ-cell exclusive genes [19,20].

Alterations in DNA methylation cooperate with genetic events in human carcinogenesis, contributing to cancer initiation, progression, invasion, and metastasis [18,19]. Aberrant hypermethylation of promoter-associated CGIs is the most common and best-characterized epigenetic abnormality in human malignancies, regardless of tissue of origin. Cancer-specific aberrant promoter hypermethylation has been established as a key mechanism for functional loss of e.g., tumor suppressor genes (TSGs), and many of the genes found to be frequently hypermethylated and epigenetically silenced in cancer cells are also commonly affected by genetic mutation or deletion [21]. In PC, aberrant DNA methylation patterns have been observed in precursor lesions and early in tumorigenesis, suggesting that epigenetic alterations may be involved in driving malignant transformation [22]. In addition, DNA methylation changes have been shown to be preserved through PC progression and metastasis, indicating that aberrant DNA methylation may function as driver event also during clonal expansion and metastatic dissemination [23]. At the molecular level, PC is a highly heterogeneous disease, which poses a major challenge for biomarker development. However, epigenetic alterations, such as promoter hypermethylation of *GSTP1*, occur more frequently and consistently than genetic mutations [24], thus suggesting that DNA methylation changes might be particularly useful as biomarkers for PC.

There are several other factors that make DNA methylation changes attractive for molecular cancer diagnostics [25,26]. First, genomic DNA is more stable than both RNA and protein, *in vivo* as well as *ex vivo* [27]. Additionally, DNA can be easily extracted from formalin-fixed and paraffin-embedded (FFPE) tissue specimens, as routinely used in the clinic e.g., for preserving diagnostic prostate needle biopsies. Moreover, contrary to most genetic mutations, DNA hypermethylation generally occurs in a particular region (promoter CGI) of a gene, potentially simplifying assay design [25]. A recent study [28] reported that although intratumor DNA methylation can be highly heterogeneous in advanced PC, methylation alterations in CGIs and at transcription start sites were relatively stable through subclonal evolution. Furthermore, DNA methylation levels can be determined by simple inexpensive methods, such as quantitative methylation-specific PCR (qMSP) that can be easily implemented into clinical practice [29]. The diagnostic potential of DNA methylation has formed the basis for the “ConfirmMDx” commercial test, which can aid PC diagnosis by detecting DNA methylation field effects in histologically benign biopsies [30]. Furthermore, it has been shown that PC cell-derived hypermethylated DNA is detectable in blood and urine samples, potentially allowing for development of non- or minimally-invasive diagnostic tests [31,32,33,34,35,36].

Multiple studies have reported highly promising potential for DNA methylation markers in relation to PC diagnosis, as recently reviewed elsewhere [37,38]. This review focuses on the utility of DNA methylation markers in relation to PC prognosis. Accordingly, we searched Pubmed using the following MESH terms: “Biological markers”, “Prostatic Neoplasms”, “DNA Methylation”, “Prognosis”, “Prognostic”, and/or “Predictive Value of Tests”. As small sample size and insufficient independent clinical validation remain major bottlenecks for translation of novel biomarkers into clinical practice, we have prioritized biomarker studies using large independent training and validation cohorts as well as robust statistical analyses, including adjustment for routine clinicopathological prognostic factors by multivariate analysis. In the following sections, we first emphasize the most extensively validated prognostic methylation marker candidates known for PC to date. Subsequently, we describe several other genes that have shown prognostic potential, but with less stringent clinical validation available at this time. Finally, to provide a more comprehensive overview, we list several additional genes that have been proposed as candidate prognostic methylation markers for PC but based on somewhat more preliminary studies.

## 2. Top Candidate Prognostic Methylation Markers for PC

This section describes the three aberrantly methylated genomic loci, *PITX2*, *C1orf114*, and *GABRE~miR-452~miR-224*, as well as the three-gene marker panel *AOX1/C1orf114/HAPLN3*, for which there is currently the strongest experimental support for a prognostic biomarker potential in PC. All candidate methylation markers described in this section have been shown to hold significant independent prognostic value in large multi-center studies including hundreds of PC patients, divided into independent training and validation cohorts. The main findings from these studies are summarized in Table 1 and further described in the text below.

### 2.1. PITX2

*PITX2* encodes the paired-like homeodomain transcription factor 2, which is induced by the Wnt/Beta-catenin pathway and involved in the regulation of cell-type specific proliferation. *PITX2* hypermethylation was initially found to correlate with postoperative disease progression in breast cancer [39] and was later found to be aberrantly hypermethylated in PC [16]. Furthermore, promoter specific hypermethylation of *PITX2* has been found to correlate with transcriptional downregulation in PC [40]. PITX2 is thought to be an upstream regulator of the insulin-like growth factor 1 receptor and of the androgen receptor, thus potentially linking it to PC initiation and progression [41]. In addition to breast and prostate cancer, aberrant hypermethylation of the *PITX2* locus has been found in acute myeloid leukemia and lung cancer [42,43].

**Table 1:** Data for the three aberrantly methylated genomic loci, *PITX2*, *C1orf114*, and *GABRE~miR-452~miR-224*, as well as the three-gene signature *AOX1/C1orf114/HAPLN3*, for which there is currently the strongest experimental support for a prognostic biomarker potential in PC. D/C: Methylation analyzed as a dichotomized/continuous variable. RP: Radical prostatectomy. FFPE: Formalin-fixed, paraffin embedded. FF: Fresh frozen; qMSP: Quantitative methylation-specific PCR; BCR: Biochemical recurrence; HR: Hazard ratio; 95% CI: 95% confidence interval; GS: Gleason score; pT: pathological tumor stage; SM: Surgical margin status; PSA: Pre-operative serum PSA.

Methylation Marker	D/C	Sample Size	Specimens	Method	End-Point	Univariate			Multivariate			Factors Adjusted for in Multivariate Analysis:	Reference
						HR	95% CI	*p*-Value	HR	95% CI	*p*-Value		
***PITX2*, training cohort**	D	585 *	RP (FFPE)	qMSP	Recurrence	3.4	1.9–6.0	<0.001	2.1	1.2–3.9	0.016	GS, pT, SM, PSA	Weiss *et al.* [16]
***PITX2*, validation cohort**	D	476	RP (FFPE)	EpiChip PITX2 Array	BCR	2.99	1.99–4.48	<0.001	2.39	1.45–3.94	<0.001	GS, pT, SM, PSA, age	Bañez *et al.* [13]
***C1orf114*, cohort 1**	C	293	RP (FFPE)	qMSP	BCR	4.74	3.00–7.48	<0.001	3.1	1.89–5.09	<0.001	GS, pT, SM, PSA, pN	Haldrup *et al.* [14]
***C1orf114*, cohort 2**	C	114	RP (FF)	qMSP	BCR	5.37	1.99–14.5	0.004	3.27	1.17–9.12	0.024	GS, pT, SM, PSA	Haldrup *et al.* [14]
***AOX1/C1orf114/HAPLN3* cohort 1**	D	293	RP (FFPE)	qMSP	BCR	2.58	1.79–3.71	<0.001	1.91	1.26–2.90	0.016	GS, pT, SM, PSA, pN	Haldrup *et al.* [14]
***AOX1/C1orf114/HAPLN3*, cohort 2**	D	114	RP (FF)	qMSP	BCR	2.64	1.55–4.51	<0.001	2.33	1.31–4.13	0.004	GS, pT, SM, PSA	Haldrup *et al.* [14]
***GABRE~miR-452~miR-224*, cohort 1**	C	293	RP (FFPE)	qMSP	BCR	1.75	1.37–2.23	<0.001	1.38	1.06–1.81	0.019	GS, pT, SM, PSA	Kristensen *et al.* [15]
***GABRE~miR-452~miR-224*, cohort 2**	C	198	RP (FF)	qMSP	BCR	2.99	1.71–5.21	<0.001	2.45	1.26–4.75	0.008	GS, pT, PSA	Kristensen *et al.* [15]

* Accurate Gleason score information missing for 239 of these samples.

The first study investigating the prognostic potential of *PITX2* promoter methylation in PC was published by Weiss and colleagues in 2009 [16]. *PITX2* methylation analysis was performed using quantitative methylation specific PCR (qMSP) on RP specimens from 585 patients with clinically localized PC from three institutions in the US. The end-point for recurrence analysis was broadly defined as either biochemical recurrence (PSA > 0.2 ng/mL on two consecutive tests), clinical recurrence, or decision to treat based on increased PSA. The authors found that high *PITX2* methylation (analyzed as a dichotomized variable, median methylation as cut-off) was significantly associated with early recurrence in both univariate Cox regression (Table 1) and Kaplan-Meier analyses [16]. Moreover, in multivariate Cox regression analysis, *PITX2* hypermethylation was shown to hold independent prognostic value when adjusting for the routine prognostic factors Gleason score, pathological tumor stage, surgical margin status, and pre-operative serum PSA (Table 1). Of note, pre-operative PSA and surgical margin status failed in the multivariate analysis. A limitation of this study was the lack of reliable information regarding Gleason score for 41% of the patients. However, multivariate analysis performed on the remaining 356 patient samples with confirmed Gleason score still showed significant independent prognostic value of *PITX2* hypermethylation beyond clinicopathological variables [16].

In a later study published by Bañez and colleagues in 2010 [13], the prognostic potential of *PITX2* was successfully validated in an independent multicenter cohort counting 476 RP patients from the US and Netherlands. Notably, the end-point in the validation study was more stringent than in the training study, namely BCR defined as PSA > 0.2 ng/mL on two consecutive tests. Furthermore, while the original training study [16] used qMSP for quantification of *PITX2* methylation, the validation study [13] used the newly developed EpiChip PITX2 Affymetrix® microarray. Thus, to allow direct comparison, a subset of 157 RP specimens from the training sample set were initially re-analyzed on the array, confirming the prognostic value of *PITX2* using the new technique. Moreover, this re-analysis was used to transfer the methylation cut-point for dichotomization to the new platform [44]. In the validation study [13], microarray data showed that high *PITX2* methylation was significantly associated with early recurrence after prostatectomy in univariate analysis, and remained significant also in multivariate analysis together with Gleason score, tumor stage, and surgical margin status, whereas pre-operative PSA showed borderline significance.

More recently, with the aim to ease future implementation into the clinic, the same group of authors developed a new qMSP assay for *PITX2* [45], which was tested using 157 RP samples from the original training study by Weiss *et al.* [16,44] and successfully validated in an independent cohort of 523 RP samples, including all 476 patients from the original validation study by Bañez *et al.* [13]. There was a very good correlation between analyses performed on the EpiChip PITX2 Affymetrix^®^ array and the new qMSP assay [45]. In summary, PITX2 hypermethylation has been found to be an independent adverse prognostic factor for recurrence after radical prostatectomy in two large independent PC patient cohorts, whether analyzed by qMSP [16,45] or using the EpiChip PITX2 Affymetrix^®^ array [13,44]. The new qMSP assay is under development for commercial use by Epigenomics AG (http://www.epigenomics.com).

### 2.2. C1orf114

To screen for new PC methylation marker candidate genes, Haldrup and colleagues [14] performed genome-wide methylation analysis of nine nonmalignant and nine PC tissue samples using the Illumina Infinium HumanMethylation27 BeadChip (Illumina 27K array), quantifying DNA methylation for 27,578 CpG sites spanning 14,495 genes. Out of hundreds of genes found to be significantly hypermethylated in PC compared to nonmalignant prostate tissue samples, the authors selected six genes (*AOX1*, *C1orf114*, *GAS6*, *HAPLN3*, *KLF8*, and *MOB3B*) for further investigation. After confirming the diagnostic potential of the six genes in a large patient cohort, their potential prognostic value was assessed using qMSP on specimens from two independent sample sets. The training set consisted of 293 RP specimens from PC patients collected in Denmark and Switzerland, whereas the validation cohort consisted of 114 RP specimens from PC patients collected in Germany and Finland. The end-point for both cohorts was BCR. For Danish, Swiss, and German patients, BCR was defined as PSA ≥ 0.2 ng/mL, whereas for Finnish patients (Cohort 2), it was PSA ≥ 0.5 ng/mL. When analyzed as a continuous variable, *C1orf114* methylation was found to be significantly associated with BCR in multivariate analysis in both cohorts, and thus identified and validated as a significant independent predictor of PSA recurrence after RP (Table 1). *C1orf114*, also known as *CCDC181*, encodes coiled-coil domain containing 181, a protein of unknown function. Notably, methylation of this gene was also significant when analyzed as a dichotomized variable [14].

Haldrup and colleagues proceeded to develop a three-gene prognostic methylation signature by combining the dichotomized methylation status of *C1orf114*, *AOX1*, and *HAPLN3* [14]. The protein encoded by *AOX1* (aldehyde oxidase 1) is involved in toxic compound metabolism, while the protein encoded by *HAPLN3* (hyaluronan and proteoglycan link protein 3) may have a function in cell adhesion. Analysis of publicly available data sets showed that both AOX1 and C1orf114 are transcriptionally downregulated in PC, consistent with the observed hypermethylation [14], whereas no significant association between methylation and expression levels was found for HAPLN3. The *AOX1/C1orf114/HAPLN3* methylation panel classified patients into low- and high-methylation subgroups, which provided independent prognostic value beyond established clinicopathological parameters in multivariate analysis in both the training cohort and in the independent validation cohort (Table 1). This three-gene methylation signature performed better than any dichotomized single gene or two-gene signature, thus showing added robustness by combining multiple markers [14].

### 2.3. GABRE~miR-452~miR-224

The *GABRE* gene encodes the epsilon subunit of the gamma-aminobutyric acid (GABA) A receptor 2, a protein whose function in benign and malignant prostatic cells has yet to be elucidated. The gene also harbors two intronic microRNAs, miR-224 and miR-452.

In a recent study by Kristensen * et al.* [15], highly cancer-specific aberrant promoter hypermethylation and significantly downregulated expression levels were observed in PC compared to nonmalignant prostate tissue, indicating that aberrant promoter hypermethylation is associated with coordinated downregulation of the entire *GABRE~miR-452~miR-224* locus in PC. In order to investigate the potential prognostic value of this locus, the methylation status of the *GABRE~miR-452~miR-224* promoter associated CGI was examined using qMSP in two independent cohorts, counting 293 RP specimens from Denmark and Switzerland, and 198 RP specimens from Germany, Finland and Sweden, respectively [15]. The end-point for both cohorts was BCR, defined as PSA ≥ 0.2 ng/mL for Danish, Swiss, Swedish, and German samples, and PSA ≥ 0.5 ng/mL for the Finnish samples. Uni- and multivariate analyses showed that high *GABRE~miR-452~miR-224* promoter methylation was significantly associated with BCR after RP in both the training and the validation cohort after adjusting for routine clinicopathological prognostic factors, whether methylation was analyzed as a continuous (Table 1) or dichotomized variable [15]. The use of two large independent cohorts and sound statistical analyses indicate that hypermethylation of the *GABRE~miR-452~miR-224* locus is a significant independent prognostic predictor of biochemical recurrence in PC patients treated by RP. Moreover, this study provided a mechanistic link between promoter hypermethylation and gene silencing, and also showed that induction of miR-452 and miR-224 inhibited proliferation, migration, and invasion of prostate cancer cell lines [15].

## 3. Emerging Candidate Methylation Markers for PC Prognosis

The candidate methylation markers described in this section have all been investigated in multiple studies, but so far with limited independent validation and/or conflicting evidence reported regarding their prognostic value. Key results from published studies of these candidates are listed in Table 2 and described in more detail below.

### 3.1. APC

This gene encodes the adenomatous polyposis coli (APC) protein, which is a well-characterized tumor suppressor regulating canonical Wnt signaling that is essential for tumorigenesis [46]. The *APC* promoter region has been found to be frequently hypermethylated in PC [47].

Through our Pubmed search, we found a total of nine studies examining the correlation between *APC* methylation status in prostate tissue samples and PC disease progression [48,49,50,51,52,53,54,55,56]. In one of these studies, Richiardi *et al.* [53] reported that *APC* hypermethylation in histologically non-neoplastic prostate adjacent to PC tumor tissue was predictive of PC specific death in a cohort of 157 patients with more than 14 years follow-up. The tissue sample set consisted of both RP, transurethral resection of the prostate (TURP) and needle-biopsy specimens, and analyses were conducted using qMSP. Not only did the authors find that *APC* hypermethylation was predictive of PC specific death in univariate and multivariate analysis (although no *p*-value was reported for the latter), but also that combined high methylation of *APC* and *GSTP1* in adjacent normal tissue was associated with significantly increased risk of PC specific death (Table 2). These results suggest a possible role of hypermethylation of adjacent non-malignant tissue in PC progression. However, a limitation of this study was that multivariate analysis was conducted adjusting for Gleason score only, and that no independent validation cohort was included [53]. In an earlier study by the same group [54], *APC* methylation was investigated (using MSP) in malignant PC tissue samples from two large patient sets, counting 216 and 243 PC specimens, respectively, and consisting of RP, TURP and diagnostic needle biopsy specimens. *APC* methylation was found to be a significant predictor of PC specific death when both cohorts were combined and was borderline significant in each of the two cohorts when analyzed separately (Table 2) [54]. Thus, in these studies [53,54], *APC* hypermethylation in adjacent non-neoplastic prostate and PC tissue, respectively, was found to be predictive of PC specific death. Although this indicates that *APC* may have independent prognostic value, both studies were limited by missing clinicopathological variables other than Gleason score.

Another study [51] investigated the correlation between *APC* hypermethylation in PC tumor tissue and BCR using qMSP analysis of 219 RP specimens. In multivariate analysis, *APC* hypermethylation was a significant predictor of BCR only in the subgroup consisting of 141 patients with tumor stage pT2 (HR (95% CI): 2.174 (1.044–4.530), *p* = 0.038) [51] (see also Table 2). When the methylation status of *APC*, *TGFB2*, and *HOXD3* was combined into a panel, the authors found that high methylation of two or more genes significantly predicted PSA recurrence in multivariate analysis (Table 2) [51]. This interesting finding, however, remains to be validated in an independent patient cohort.

In concordance with the three studies described above, two smaller studies [50,55] reported *APC* hypermethylation to be significantly associated with either BCR, metastasis and/or death in multivariate analysis (Table 2). One of these studies was conducted on RP samples exclusively from patients with Gleason score 7 tumors [55], the other on needle-biopsy samples from PC patients treated by RP, RT and/or androgen deprivation therapy (ADT) [50]. In contrast to the five studies already mentioned, four studies [48,49,52,56] found no significant association between *APC* hypermethylation and PC progression, which for three of the studies [49,52,56] could be due to small sample size (<85 patients, Table 2). The last of the four negative studies [48] was conducted using MSP on 151 RP specimens, but no significant correlation was found between *APC* methylation and BCR in univariate analysis.

In summary, several studies have shown a significant correlation between *APC* hypermethylation and increased risk of PC progression, suggesting that methylation of *APC* could have prognostic utility for PC. Accordingly, further investigations are warranted for this candidate marker, especially large-scale studies using well-defined PC patient cohorts as well as clearly defined clinical endpoints.

**Table 2:** Data for methylation marker candidates subject to investigation in multiple studies, but with limited validation and/or conflicting evidence regarding their prognostic value. RP: Radical prostatectomy; TURP: Transurethral resection of the prostate; FFPE: Formalin-fixed, paraffin embedded; FF: Fresh frozen; MSP: Methylation-specific PCR; qMSP: Quantitative MSP; BCR: Biochemical recurrence; HR: Hazard ratio; OR: Odds ratio; 95% CI: 95% confidence interval; NA: Not available; GS: Gleason score; pT: pathological tumor stage; SM: Surgical margin status; PSA: Pre-operative serum PSA; EP: Extracapsular penetration; pN: Pathological lymph node status; SV: Seminal vesicles involvement.

Methylation Marker	Sample Size	Specimens	Method	End-Point	Univariate			Multivariate			Factors Adjusted for in Multivariate Analysis:	Reference
					HR	95% CI	*p*-Value	HR	95% CI	*p*-Value		
***ABHD9***	223	RP (FFPE)	qMSP	BCR	NA	NA	<0.001	NA	NA	0.016	GS, pT, SM	Cottrell *et al.* [57]
***ABHD9***	592	RP (FFPE)	qMSP	BCR/Clinical recurrence	1.9	1.1–3.1	0.02	NA	NA	NA	NA	Weiss *et al.* [16]
***ABHD9***	407	RP (FFPE)	450K	Progression/PC specific death	NA	NA	NA	1.16	0.77–1.75	NA	Age, GS, pT, PSA	Stott-Miller *et al.* [58]
***APC***	157	Benign tissue (RP, TURP, needle biopsies) (FFPE)	qMSP	PC specific death	NA	NA	0.007	1.91	1.03–3.56	NA	GS, methylation in tumor tissue	Richiardi *et al.*, 2013 [53]
***APC/GSTP1***	157	Benign tissue (RP, TURP, needle biopsies) (FFPE)	qMSP	PC specific death	NA	NA	NA	2.4	1.15–5.01	0.032	GS, methylation in tumor tissue	Richiardi *et al.*, 2013 [53]
***APC*, cohort 1 + 2**	459	RP, TURP, needle biopsies (FFPE)	MSP	PC specific death	NA	NA	NA	1.49	1.11–2.00	NA	GS	Richiardi *et al.*, 2009 [54]
***APC*, cohort 1**	216	RP, TURP, needle biopsies (FFPE)	MSP	PC specific death	NA	NA	0.11	1.42	0.98–2.07	NA	GS	Richiardi *et al.*, 2009 [54]
***APC*, cohort 2**	243	RP, TURP, needle biopsies (FFPE)	MSP	PC specific death	NA	NA	0.02	1.57	0.95–2.62	NA	GS	Richiardi *et al.*, 2009 [54]
***APC***	219	RP (FFPE)	qMSP	BCR	NA	NA	0.028	2.22	0.78–6.32	0.137	GS, pT, SM, age	Liu *et al.* [51]
***APC/TGFB2/HOXD3***	219	RP (FFPE)	qMSP	BCR	NA	NA	<0.001	2.01	1.14–3.57	0.017	GS, pT, SM, age	Liu *et al.* [51]
***APC***	74	RP (GS 7) (FFPE)	qMSP	BCR	1.6	0.8–3.19	0.18	3	1.42–6.32	0.004	Age, GSTP1 hypermethylation	Rosenbaum *et al.* [55]
***APC***	83	Needle biopsies	qMSP	PC specific survival	NA	NA	0.01	3.51 (OR)	1.23–9.96	0.018	Age, PSA, pT, GS	Henrique *et al.* [50]
***APC***	83	Needle biopsies	qMSP	BCR	NA	NA	0.002	2.58 (OR)	1.29–5.16	0.008	Age, PSA, pT, GS	Henrique *et al.* [50]
***APC***	151	RP (FFPE)	MSP	BCR	1.26 (OR)	0.58–2.74	0.57	NA	NA	NA	NA	Alumkal *et al.* [48]
***APC***	84	RP (GS ≤ 7) (FFPE)	qMSP	BCR	0.667	0.21–2.15	0.497	NA	NA	NA	NA	Moritz *et al.* [52]
***Chr3-EST***	223	RP (FFPE)	qMSP	BCR	NA	NA	<0.001	NA	NA	0.043	GS, pT, SM	Cottrell *et al.* [57]
***Chr3-EST***	598	RP (FFPE)	qMSP	BCR/Clinical recurrence	2.1	1.2–3.5	0.007	NA	NA	NA	NA	Weiss *et al.* [16]
***GSTP1***	157	Benign tissue (RP, TURP, needle biopsies) (FFPE)	qMSP	PC specific death	NA	NA	0.02	1.6	0.80–3.19	NA	GS, methylation in tumor tissue	Richiardi *et al.*, 2013 [53]
***GSTP1***	83	Needle biopsies	qMSP	BCR	NA	NA	0.047	NA	NA	NA	NA	Henrique *et al.* [50]
***GSTP1***	74	RP (GS 7) (FFPE)	qMSP	BCR/metastasis/death	0.34	0.13–0.88	0.03	0.29	0.11–0.77	0.01	Age, APC/CCND2 hypermethylation	Rosenbaum *et al.* [55]
***GSTP1***	151	RP (FFPE)	MSP	BCR	1.07 (OR)	0.53–2.18	0.85	0.30 (OR)	0.07–1.24	0.1	GS, SM, SV, EP, PSA, pN	Alumkal *et al.* [48]
***GSTP1***	84	RP (GS ≤ 7) (FFPE)	qMSP	BCR	1.772	0.76–4.15	0.187	NA	NA	NA	NA	Moritz *et al.* [52]
***GSTP1*, cohort 1**	216	RP, TURP, needle biopsies (FFPE)	MSP	PC specific death	NA	NA	NA	1	0.64–1.58	NA	GS	Richiardi *et al.*, 2009 [54]
***GSTP1*, cohort 2**	243	RP, TURP, needle biopsies (FFPE)	MSP	PC specific death	NA	NA	NA	1.44	0.82–2.54	NA	GS	Richiardi *et al.*, 2009 [54]
***GSTP1***	60	RP	qMSP	BCR	5.31 (OR)	0.63–45.07	0.13	NA	NA	NA	NA	Woodson *et al.*, 2006 [59]
***HOXD3***	232	RP	qMSP	BCR	NA	NA	0.043	0.5	0.19–1.33	0.16	GS, SM, pT	Kron *et al.*, 2010 [60]
***HOXD3***	195	RP	qMSP	BCR	NA	NA	0.067	1.246	0.72–2.15	0.431	pT, SM, age, GS, PSA	Kron *et al.*, 2012 [61]
***HOXD3***	407	RP (FFPE)	450K	Progression/PC specific death	NA	NA	NA	1.7	1.14–2.54	NA	Age, GS, pT, PSA	Stott-Miller *et al.* [58]
***PTGS2***	36	RP (FF)	qMSP	BCR	2.82	1.07-7.44	0.04	4.26	1.36–13.36	0.01	GS, pT, PSA	Yegnasubramanian *et al.* [56]
***PTGS2/CD44***	60	RP	qMSP	BCR	10.56 (OR)	2.35–47.54	0.002	8.87 (OR)	1.85–42.56	0.006	GS	Woodson *et al.*, 2006 [59]
***PTGS2***	60	RP	qMSP	BCR	4.38 (OR)	1.13–17.40	0.04	NA	NA	NA	NA	Woodson *et al.*, 2006 [59]
***RARB***	84	RP (GS ≤ 7) (FFPE)	qMSP	BCR	2.686	1.15–6.29	0.023	1.674	0.69-4.06	0.254	GS, pT, PSA, pN,	Moritz *et al.* [52]
***RARB***	74	RP (GS 7) (FFPE)	qMSP	BCR/metastasis/death	1.22	0.59–2.52	0.59	NA	NA	NA	NA	Rosenbaum *et al.* [55]
***RARB***	60	RP	qMSP	BCR	3.34 (OR)	0.66–17.29	0.14	NA	NA	NA	NA	Woodson *et al.*, 2006 [59]
***RASSF1A***	83	Needle biopsies	qMSP	BCR	NA	NA	0.019	NA	NA	NA	NA	Henrique *et al.* [50]
***RASSF1A***	219	RP (FFPE)	qMSP	BCR	NA	NA	0.556	0.74	0.42–1.29	0.284	GS, pT, SM, age	Liu *et al.* [51]
***RASSF1A***	74	RP (GS 7) (FFPE)	qMSP	BCR/metastasis/death	0.7	0.31–1.59	0.39	NA	NA	NA	NA	Rosenbaum *et al.* [55]

### 3.2. PTGS2

*PTGS2* encodes prostaglandin-endoperoxide synthase 2, or cyclooxygenase-2 (COX-2), a pro-inflammatory enzyme required for prostaglandin biosynthesis. It promotes carcinogenesis by stimulating cell growth, survival, invasiveness, and neoangiogenesis, and high expression of *PTGS2* has been associated with adverse clinical outcome in several human hematological malignancies and solid tumors, including PC [62]. In seeming conflict with this, however, several studies have reportedpromoter hypermethylation of *PTGS2* in PC [49,63,64]. Hypermethylated *PTGS2* has also been detected in body fluid samples from PC patients (discussed below).

The correlation between *PTGS2* methylation in RP specimens from PC patients and BCR has been investigated in three studies [49,56,59], all using qMSP on samples from 60 patients or less. In one study [56], including only 36 patients, high methylation of *PTGS2* was found to indicate a more than four-fold increased risk of BCR after adjusting for Gleason score, tumor stage, and pre-operative PSA in multivariate analysis (Table 2). Another study [59] found that the combined hypermethylation of *PTGS2* and *CD44* gave an almost nine-fold increased risk of BCR in multivariate analysis adjusting for Gleason score only (Table 2), although in this study, *PTGS2* methylation alone was significant in univariate analysis only (Table 2) [59]. Finally, a single study [49] found no significant correlation between *PTGS2* methylation status and BCR. Accordingly, further investigation in larger cohorts is needed to assess the potential prognostic value of *PTGS2* methylation in PC and, furthermore, how this may relate to gene expression.

### 3.3. RARB

*RARB* encodes the retinoic acid receptor beta, a nuclear transcriptional regulator mediating cellular signaling in cell growth and differentiation. It is expressed in most tissues and is thought to exert a tumor suppressor function by regulating gene expression. Furthermore, *RARB* is frequently found silenced and hypermethylated in PC [65].

In total, five studies have investigated methylation of *RARB* in RP or needle-biopsy specimens, all using sample sets counting <100 specimens, various clinical end-points, and either quantitative or non-quantitative MSP [49,50,52,55,59]. Only one study [52] reported significant association between *RARB* hypermethylation and BCR in a sample set of 84 RP specimens from tumors with Gleason score ≤7, where *RARB* hypermethylation was a significant predictor of BCR in univariate Cox regression analysis when analyzed as a continuous variable, and also in Kaplan-Meier analysis as a dichotomized variable. It was, however, not found significant in multivariate analysis (Table 2) [52].

Thus, so far, the correlation between *RARB* methylation and PC progression has only been subject to investigation in small sample sets, and further studies examining the association to PC prognosis in large patient cohorts are needed.

### 3.4. RASSF1A

This gene encodes the RAS association domain-containing protein 1, which exerts classic tumor suppressor functions through its involvement in DNA repair and induction of cell cycle arrest. Loss or altered expression of RASSF1A has been found in many cancers, and gene silencing has been found correlated with promoter-associated CGI hypermethylation in PC [66].

A total of four studies have investigated the correlation between *RASSF1A* methylation status in either RP or needle-biopsy specimens and risk of PC progression (various clinical endpoints) using qMSP [50,51,55,56]. Small sample size was a limiting factor in three of the four studies [50,55,56]. In one of the smaller studies [50], *RASSF1A* hypermethylation was investigated in 83 needle-biopsy specimens from PC patients treated by RP, RT, and/or ADT. A significant association between *RASSF1A* hypermethylation and BCR was found in univariate log-rank, but not in multivariate analysis (Table 2) [50]. The only study using a larger cohort, counting 219 PC patients [51], did not find a significant correlation between *RASSF1A* methylation status and BCR (Table 2), nor did the remaining two small-scale studies [55,56].

So far, rather preliminary results have been reported for *RASSF1A* methylation in relation to PC progression, and further studies are needed to investigate whether *RASSF1A* hypermethylation adds independent prognostic value to the established clinicopathological parameters.

### 3.5. ABHD9

*ABHD9*, or *EPHX3*, encodes epoxide hydrolase 3. Little is known about this protein, except that it is involved in detoxification and processing of epoxides. The *ABHD9* promoter region was found to be significantly hypermethylated in high grade PC in a genome-wide screening study using a methylation microarray for analysis of 304 PC tumor specimens [57]. The authors tested these findings in a new independent patient sample set of 223 RP specimens using qMSP [57]. Here, *ABHD9* hypermethylation was found to be predictive of BCR in univariate as well as multivariate analysis, adjusting for Gleason score, tumor stage, and surgical margin status (Table 2). Another study [16], using qMSP on 592 RP specimens, reported a significant association between *ABHD9* hypermethylation and PC progression (BCR or clinical recurrence) in univariate, but not in multivariate analysis (Table 2). Finally, a more recent study [58] investigated the methylation status in RP specimens from 407 PC patients using the Illumina Infinium HumanMethylation450 BeadChip (Illumina 450K array), which interrogates >450,000 CpG sites in the human genome. Here, *ABDH9* hypermethylation was not found to be significantly associated with time to progression (Table 2), however in this study, progression was very broadly defined as either BCR (PSA ≥ 0.2 ng/mL), receipt of secondary treatment, positive imaging results, prostate bed or lymph node biopsy showing tumor content, diagnosis of tumor recurrence, or PC-specific death.

Based on these studies, which all include large sample sizes, there is some evidence supporting *ABHD9* hypermethylation as a possible independent prognostic marker for PC and further investigation is warranted.

### 3.6. HOXD3

This gene (also known as *HOX4*, *HOX1D*, *HOX4A* and *Hox-4.1*) encodes homeobox D3, a protein that may play a role in the regulation of cell adhesion processes, but a clear function has yet to be elucidated. In PC, the *HOXD3* promoter region has been found hypermethylated [60,67], but there is no clear indication of differential HOXD3 expression between benign and malignant prostate tissue according to publicly available datasets [68].

By methylation analysis of 232 RP specimens using qMSP [60], Kron and colleagues found that *HOXD3* was a significant independent predictor of BCR in univariate log-rank analysis, but not in multivariate Cox regression analysis adjusting for Gleason score, tumor stage, and surgical margin status (Table 2). However, when analyzing the combined methylation status of *HOXD3*, *APC*, and/or *TGFB2*, high methylation was shown to be predictive of BCR in multivariate analysis [51]. Furthermore, a more recent study by Kron *et al.* [61] failed to confirm the significant association between *HOXD3* methylation and BCR in univariate as well as multivariate analysis of 195 RP specimens using qMSP (Table 2).

The Illumina 450K methylation array was recently used to analyze methylation of 407 RP specimens, using a broad spectrum of clinical end points for progression [58]. When analyzed as a dichotomized variable, *HOXD3* methylation was found significantly associated with progression in univariate log-rank analysis as well as in multivariate analysis adjusting for age, Gleason score, pre-operative PSA, and pathological tumor stage (Table 2). However, the median time for progression-free survival was very long in the low (19.4 years) as well as in the high methylation group (17.0 years) [58].

Thus, based on these analyses, there is some evidence supporting a prognostic value of *HOXD3* methylation, but further investigation is needed.

### 3.7. Chr3-EST

*Chr3-EST*, an expressed sequence tag on chromosome 3, was reported as hypermethylated in high grade tumors in a genome-wide screening study using a methylation microarray for profiling of 304 RP tumor specimens [57]. Further investigation, using qMSP on an independent sample set of 223 RP specimens, showed that hypermethylated *Chr3-EST* was a borderline significant predictor of BCR in univariate log-rank analysis, and also significant in multivariate Cox regression analysis adjusting for Gleason score, tumor stage, and surgical margin status (Table 2) [57]. However, another study [16] investigating *Chr3-EST* methylation in 598 RP specimens using qMSP, reported that *Chr3-EST* was a significant predictor of biochemical or clinical recurrence in univariate analysis only (Table 2). These results from two studies using large patient cohorts provide some preliminary evidence of a prognostic potential of *Chr3-EST* methylation in PC and thus warrant further investigations.

### 3.8. GSTP1

*GSTP1* encodes glutathione *S*-transferase, a tumor suppressing enzyme involved in drug metabolism and detoxification, working to protect DNA from oxidative damage. Hypermethylation of the *GSTP1* promoter-associated CGI is the most extensively studied epigenetic biomarker for PC, particularly with regards to diagnostic applications [69]. *GSTP1* hypermethylation has been observed in >90% of PC tissues, but is rarely seen in the histologically normal prostate or in other human tissues, occurring with significant frequency only in liver and breast cancers [56,70]. Thus, *GSTP1* hypermethylation has been suggested as a diagnostic PC biomarker in tissue as well as body fluids.

A total of nine studies have investigated *GSTP1* promoter methylation in either adjacent non-malignant or prostate cancer tissue samples in relation to risk of PC recurrence by multivariate analysis. First, Richiardi *et al.* [53] investigated *GSTP1* hypermethylation in 157 samples of non-malignant tissue adjacent to tumor from RP, TURP, or needle-biopsy specimens using qMSP. In this study, *GSTP1* methylation was significantly correlated to PC specific death in univariate analysis, but *GSTP1* was not independent of Gleason score in multivariate analysis (Table 2) unless combined with *APC*, as mentioned above (Table 2) [53].

Furthermore, a smaller study [50] investigated *GSTP1* methylation in malignant tissue samples from 83 diagnostic needle-biopsy specimens from PC patients treated by RP, RT and/or ADT, and showed a moderate correlation between *GSTP1* hypermethylation and time to BCR in log-rank univariate analysis, but no significance in multivariate Cox regression analysis (Table 2). Another small-scale study [55] analyzed *GSTP1* methylation in 74 RP specimens from PC tumors with Gleason score 7. Here, patients with high levels of *GSTP1* methylation in fact were found to have significantly *decreased* risk of progression (BCR, metastasis, or death) after RP as compared to the low-methylation group in multivariate analysis (Table 2) [55]. The remaining six studies reported no significant correlation between *GSTP1* methylation status in malignant tissue samples and PC progression [48,49,52,54,56,59]. Amongst these was a large scale study [54] comprising two large independent sample sets counting 216 and 243 RP, TURP, and needle biopsy PC tumor specimens, respectively, reporting no significant correlation between *GSTP1* methylation and PC specific death (Table 2). Taken together, conflicting evidence has been published regarding the prognostic value of *GSTP1* hypermethylation, and further investigations are needed.

## 4. Other Genes Investigated as Potential Methylation Markers for PC Prognosis

Table 3 lists several other genes that have been investigated as prognostic candidate methylation biomarkers for PC in at least two cohorts, and for which statistically significant correlation between methylation levels and PC progression was found in univariate but not in multivariate analysis. Table 3 also lists a number of candidate methylation markers that have shown prognostic potential in only a single patient cohort so far.

Amongst the listed candidate genes is *GPR7* (also known as *NPBWR1*). This gene encodes neuropeptide B/W receptor 1, a protein with neuroendocrine function but its potential role in prostate tissue is unknown. So far, two studies have conducted methylation analysis of the *GPR7* promoter region on large sample sets counting 153 and 598 RP specimens, respectively [16,57]. In both studies, hypermethylation of *GPR7* was a significant predictor of BCR or local recurrence in univariate, but not multivariate analysis (Table 3). Likewise, aberrant promoter hypermethylation of *CD44* has been observed in PC and loss of CD44 expression has been associated with PC aggressiveness [71]. *CD44* encodes a cell-surface glycoprotein involved in cell-cell interaction, cell migration and adhesion. Hypermethylation of the *CD44* promoter region was found to be predictive of BCR in univariate analysis in a single study [59], using a small sample set of 60 RP specimens, whereas a larger study [48] of 151 RP specimens was unable to confirm this (Table 3).

Another study [72], comprising one sample set of 149 RP specimens, showed that hypermethylation and downregulation of *miR-205* was a significant predictor of PC progression in both univariate and multivariate analysis (Table 3). Moreover, another study [73] investigated the DNA methylation of *KLK6* and *KLK10* in two large RP sample sets (*n*_cohort1_ = 150, *n*_cohort2_ = 124) using qMSP. However, clinical follow-up data was not available for cohort 1 and the prognostic potential was therefore investigated in cohort 2 only, where it was found that *hypo* methylation of *KLK10* was a significant predictor of BCR in both univariate and multivariate analysis (Table 3). While these results are clearly interesting, the prognostic potential of both *miR-205* and *KLK10* has been demonstrated only in a single cohort so far. Thus, further investigations are needed to assess their potential value for PC prognosis.

## 5. Genome-Wide Methylation Profiling

In recent years, new technologies such as methylation microarrays and next-generation sequencing (NGS) have made it possible to conduct genome-wide DNA methylome profiling studies, in turn uncovering focal hypermethylation at multiple gene loci at single base resolution. Thus, biomarker discovery studies using these novel technologies for genome-wide screening of DNA methylation aberrations are now beginning to produce long lists of novel candidate methylation markers for PC diagnosis and prognosis.

In one study [74], the Illumina 27K methylation array was used to investigate DNA methylation in 238 tissue samples from PC patients. Binary analyses were conducted for PC (*n* = 198, RP specimens) *vs.* non-malignant prostate tissue samples (*n* = 40), recurrence (clinical recurrence or BCR, *n* = 123) *vs.* non-recurrence (*n* = 75), as well as for specific recurrence sub-groups (clinical *vs.* BCR, local *vs.* systemic). These analyses generated lists of several significantly differentially methylated genes, with a total of 147 genes in the comparison of PC *vs.* nonmalignant prostate tissue, 75 in the recurrence *vs.* non-recurrence analysis, 16 in the comparison of clinical *vs.* biochemical recurrence, and 68 in the local *vs.* systemic recurrence analysis. For each comparison, three to five genes were selected for validation by pyrosequencing in an independent patient set, in each case counting a total of 20 samples relevant to the groups being analyzed. Additionally, in order to more directly predict relapse and risk of BCR, uni- and multivariate Cox regression analyses were conducted [74]. In multivariate analysis, adjusting for Gleason score and tumor stage, 183 genes were found to predict PC recurrence. Here, 16 of the 75 genes found through binary analysis of recurrence *vs.* non-recurrence were reported to be among the 183 genes significant in the multivariate analysis, and three of these (*FLNC*, *HS3ST2* and *RASGRF2*) were also amongst the candidates validated by pyrosequencing [74].

Another study [75] used the more recently developed Illumina 450K methylation array to investigate global methylation patterns in a small sample set of 19 PC (RP) and four non-malignant specimens. A total of 7031 genes were identified as significantly differentially methylated between cancerous and non-malignant prostate tissue, of which 122 were also inversely correlated to expression. However, when comparing recurrent (BCR) *vs.* non-recurrent PC, only two CpG sites were significantly differentially methylated, but these were not subjected to further validation [75]. In a separate study, also using the Illumina 450K array, Stott-Miller *et al.* [58] investigated the methylation status of a set of 14 previously reported candidate methylation markers for PC prognosis (*ABHD9*, *APC*, *ASC*, *CD44*, *CDH13*, *GPR7*, *GSTP1*, *HOXD3*, *MDR1*, *PITX2*, *PTGS2*, *RARB*, *RASSF1A*, and *RUNX3*) in 407 PC specimens from RP patients. A gene was considered to be validated as a prognostic candidate marker if at least 50% of the promoter-associated CpG sites interrogated by the array were significantly hypermethylated in tumor samples from recurrent patients compared to non-recurrent patients. Thus, only *ABHD9* and *HOXD3* were deemed significant [58], as discussed above for the respective genes. However, statistical significance was the sole criteria for significant hypermethylation, whereas the absolute difference in methylation levels between the two investigated groups was not considered in this study [58].

**Table 3:** Data for genes investigated as prognostic methylation biomarkers candidates for PC in at least two cohorts, showing statistically significant correlation to PC progression in univariate analysis only, in addition to candidate methylation markers that have shown prognostic potential in a single cohort; RP: Radical prostatectomy; TURP: Transurethral resection of the prostate; FFPE: Formalin-fixed, paraffin embedded; FF: Fresh frozen; MSP: Methylation-specific PCR; qMSP: Quantitative MSP; BCR: Biochemical recurrence; HR: Hazard ratio; OR: Odds ratio: 95% CI: 95% confidence interval; NA: Not available; GS: Gleason score; pT: pathological tumor stage; SM: Surgical margin status; PSA: Pre-operative serum PSA; EP: Extracapsular penetration; SV: Seminal vesicle involvement; pN: Pathological lymph node status.

Methylation Marker	Sample Size	Specimens	Method	End-Point	Univariate			Multivariate			Factors Adjusted for in Multivariate Analysis:	Reference
					HR	95% CI	*p*-Value	HR	95% CI	*p*-Value		
***AIM1***	95	RP (GS 7) (FFPE)	qMSP	BCR	0.4	0.18–0.89	0.02	0.45	0.2–1.0	0.05	pN, age	Rosenbaum *et al.* [55]
***CD44***	60	RP	qMSP	BCR	6.83 (OR)	1.67–27.99	0.008	NA	NA	NA	NA	Woodson *et al.*, 2006 [59]
***CDH13***	151	RP (FFPE)	MSP	BCR	1.80 (OR)	0.90–3.61	0.1	5.51 (OR)	1.34–22.67	0.02	GS, SM, SV, EP, PSA	Alumkal *et al.* [48]
***CDH13/ASC***	151	RP (FFPE)	MSP	BCR	NA	NA	NA	5.64 (OR)	1.47–21.7	0.01	GS, SM, SV, EP, PSA	Alumkal *et al.* [48]
***CDKN2A***	151	RP (FFPE)	MSP	BCR	0.43 (OR)	0.19–0.98	0.05	0.43 (OR)	0.10–1.90	0.27	GS, SM, SV, EP, PSA	Alumkal *et al.* [48]
***GPR7***	596	RP (FFPE)	qMSP	BCR/clinical recurrence	2.3	1.4–3.9	0.002	NA	NA	NA	NA	Weiss *et al.* [16]
***GPR7***	153	RP (FFPE)	Methylation array	BCR	NA	NA	0.0002	NA	NA	NA	NA	Cottrell *et al.* [57]
***HSBP1***	415	Needle biopsies, TURP (FF/FFPE)	Pyro-sequencing	PC specific death	1.12	1.02–1.23	0.02	1.18	0.98–1.41	0.075	GS, PSA, % PC in biopsy, age	Vasiljevic *et al.* [76]
***KLK10***	124	RP	qMSP	BCR	NA	NA	0.046	2.11	1.10–4.02	0.028	GS, pT, SM	Olkhov-Mitsel *et al.* [73]
***miR-205***	149	RP (FF)	qMSP	BCR or local recurrence	2.62	1.2–5.7	0.012	2.23	0.99–5.0	0.05	GS, pT	Hulf *et al.* [72]
***RUNX3* (cohort 1)**	216	RP, TURP, needle biopsy (FFPE)	MSP	PC specific death	NA	NA	0.32	1.22	0.70–2.14	NA	GS	Richiardi *et al.*, 2009 [54]
***RUNX3* (cohort 2)**	243	RP, TURP, needle biopsy (FFPE)	MSP	PC specific death	NA	NA	0.05	1.56	0.95–2.56	NA	GS	Richiardi *et al.*,2009 [54]
***SOCS3***	35	RP (FFPE)	MSP	BCR/local recurrence/metastasis	13.01	2.58–65.47	0.0019	11.17	1.288–96.96	0.029	GS, pT, PSA, SM	Pierconti *et al.* [77]
***TBX15***	195	RP	qMSP	BCR	NA	NA	0.003	1.241	0.731–2.104	0.424	pT, SM, age, GS, PSA	Kron *et al.*, 2012 [61]
***TBX15/HOXD3***	195	RP	qMSP	BCR	NA	NA	0.002	1.409	0.864–2.299	0.17	pT, SM, age, GS, PSA	Kron *et al.*, 2012 [61]

In a recent NGS study, Lin *et al.* [78] used enhanced reduced representation bisulfite sequencing (eRRBS) to investigate DNA methylation at >2.5 million single CpG sites in seven matched pairs of PC and non-malignant tissue samples as well as six CRPC specimens with neuroendocrine phenotype, leading to the identification of a panel of 13 gene-associated CGIs (*GSTP1*, *GRASP*, *TMP4*, *KCNC2*, *TBX1*, *ZDHHC1*, *CAPG*, *RARRES2*, *SAC3D1*, *NKX2-1*, *FAM107A*, *SLC13A3*, *FILIP1L*) that exhibited increased methylation in disease progression concurrent with downregulation of expression. The ability of the 13-gene panel to distinguish non-malignant prostate tissue from PC, as well as localized PC from CRPC, was confirmed in a small independent patient set. While these results are interesting, further studies in large PC patient cohorts with long follow-up are needed to assess the actual clinical value of these 13 CGIs as potential diagnostic and/or prognostic biomarkers [78].

In summary, the development of new technologies for genome-wide methylation analysis has led to the identification of a vast number of genes aberrantly methylated in PC and thereby has accelerated the discovery of novel candidate biomarkers for PC diagnosis and prognosis. However, most of the studies published so far have been conducted using relatively small discovery cohorts and/or lack sufficient independent clinical validation. Furthermore, it largely remains to be investigated to which extent aberrant methylation patterns in genomic regions outside of gene promoters and outside of CGIs might be used for biomarker development in PC.

## 6. DNA Methylation in Biofluids

DNA methylation of circulating cell-free DNA (cfDNA) is detectable in body fluids, including whole blood, plasma, serum, urine, and semen [36,79,80], and thus holds promise for future clinical use as it may enable detection of tumor-specific epigenetic aberrations by non-invasive testing*.* Moreover, non-invasive biomarkers can be analyzed repeatedly in the same patient, potentially allowing for real-time monitoring of e.g*.*, disease progression and therapy response. A meta-analysis of 22 peer-reviewed studies concluded that *GSTP1* hypermethylation has great potential as a body fluid biomarker for PC diagnosis [80]. Indeed, detection of *GSTP1* hypermethylation in plasma, serum and/or urine samples was found to predict PC with much higher specificity (>90%) than PSA (approximately 20%), although the sensitivity of *GSTP1* was modest [80]. In contrast, few studies have investigated the prognostic potential of measuring DNA methylation in biofluids.

Bastian and colleagues [31] used qMSP to investigate the association between BCR and *GSTP1* methylation in pre-operative serum samples from 85 PC patients treated by RP. In univariate analysis, *GSTP1* hypermethylation was associated with 24-fold increased risk of BCR (HR (95% CI): 24.0 (2.5–231.2); *p* = 0.006), but only four patients suffered BCR in this cohort. Therefore, *GSTP1* hypermethylation was investigated in pre-operative serum samples from a larger cohort of 110 PC patients of which 50% suffered BCR after RP [31]. Of note, the two sub-groups in this second cohort were matched for Gleason score. Consistent with the initial findings, *GSTP1* hypermethylation was found to be significantly associated with BCR in univariate (HR (95% CI): 3.0 (1.4–6.4); *p* < 0.001) as well as in multivariate analysis, where hypermethylated *GSTP1* in serum was associated with 4.4-fold increased risk of BCR (HR (95% CI): 4.4 (2.2–8.8), *p* < 0.001) [31]. However, in another study using qMSP analysis, Ellinger and colleagues [32] found no significant association between BCR and detection of hypermethylated *GSTP1* (or of *PTGS2*, *Reprimo*, or *TIG1*) in pre-operative serum samples from 122 PC patients treated by RP [32].

Another study [33] analyzed whole blood samples from 76 CRPC patients, treated by maximum androgen blockage, in order to detect circulating tumor cells (CTCs) and methylated DNA (using qMSP) for a five-gene candidate marker panel (*GSTP1*, *APC*, *PTGS2*, *MDR1* and *RASSF1A*). Here, detection of CTCs was highly significantly correlated with detection of DNA methylation, suggesting that CTCs contribute genomic DNA to the methylation analysis. Detection of at least one methylated marker gene (*vs.* none) in whole blood was highly statistically significant for poor overall survival in univariate (*p* < 0.001) as well as multivariate analysis (HR (95% CI) of 1.83 (1.07–2.96), *p* < 0.001). Detection of CTCs also remained significant in the multivariate model (*p* = 0.019), whereas Gleason score and pre-operative PSA failed. The prognostic potential of individual marker genes was not reported [33].

Future prognostic tests for PC may also be urine-based. An early study [36] reported that *GSTP1* hypermethylation was detectable by qMSP in urine specimens after prostatic massage with high sensitivity and specificity for PC. Furthermore, Payne and colleagues [34] reported that all four investigated PC methylation marker candidates in their study, including *GSTP1*, performed better in qMSP analysis of urine compared to plasma samples, raising the possibility that urine samples may be particularly useful for detection of PC tumor-specific DNA methylation. As proof of principle, these preliminary studies show that detection of aberrant cancer-specific DNA methylation in biofluids is feasible and may be useful for development of clinically relevant prognostic biomarkers for PC.

## 7. Discussion

The routine prognostic indicators available today cannot distinguish clearly between aggressive and non-aggressive PC at the time of diagnosis [81]. Combined with excessive use of PSA testing for early detection, this has led to overdiagnosis and overtreatment of many clinically insignificant PCs [82]. Accordingly, there is an urgent need for novel PC biomarkers that can pave the way for better and more personalized treatment.

Several published studies have shown that aberrant focal DNA methylation has the potential to make excellent biomarkers for early cancer detection as well as prediction of outcome [37,38,83,84]. DNA methylation analysis may also increase the sensitivity of PC diagnosis upon biopsy, as aberrant methylation has been detected in histologically normal prostate biopsy specimens from patients with a subsequent cancer diagnosis [30,85,86]. Moreover, PC cell-derived aberrant DNA methylation is detectable in body fluids, making non-invasive testing a possibility [36,79,80]. Several studies have shown that DNA methylation levels correlate with clinicopathological markers of aggressiveness, such as Gleason score and tumor stage [51,52,57,60,61,77,87,88]. Thus, classification based on methylation profiles alone, or in combination with routine clinicopathological variables, could be useful for predicting the clinical course of PC and thus guide treatment decisions [26]. We note that most of the prognostic methylation marker candidates described in this review have been reported to be highly cancer-specific and thus hold potential also as diagnostic biomarker candidates for PC (for recent review see e.g., [37,38]).

Of the methylation marker candidates investigated to date, *PITX2*, *C1orf114*, *GABRE~miR-452~miR-224* and the three-gene panel *AOX1/C1orf114/HAPLN3* currently hold the strongest experimental support for independent prognostic biomarker potential beyond routine clinicopathological variables. The prognostic potential of these candidates for prediction of BCR after RP has been demonstrated in retrospective studies characterized by careful experimental design, including the use of large multi-center PC patient cohorts, stringent independent clinical validation, and multivariate analysis adjusting for routine clinicopathological prognostic factors [13,14,15,16]. Of note, *GABRE~miR-452~miR-224* and *C1orf114* were found to be highly significant in multivariate analysis not only as dichotomized variables but also as continuous variables, in further support of their potential as independent prognostic biomarkers (see below). Furthermore, combining *C1orf114* with two other genes (*AOX1* and *HAPLN3*) in a dichotomized methylation signature was found to increase the prognostic value [14]. Several other studies have also reported that the combination of several biomarker candidates into “signatures” or “panels” has the potential to improve specificity as well as sensitivity, compared to a single biomarker [51,53,55,59,61]. The combination of multiple markers may be particularly useful for PC, which is a notoriously highly heterogeneous disease at the molecular level [89].

The prognostic potential for the remaining candidate biomarkers discussed in this review is supported only by more preliminary evidence. These candidates showed significant prognostic potential in some studies, but not in others, or completely lacked validation. The conflicting findings may be explained by variations in experimental setup and study designs, such as differences in patient cohorts, methods for methylation analysis, sample type and processing, combined with the use of rather small cohorts with limited follow-up in some of the studies. Moreover, we found that many studies did not report results from multivariate analysis unless statistically significant, and there was great variability as to which extent the studies adjusted for known prognostic factors, including tumor stage, Gleason score, pre-operative serum PSA, surgical margin status, and ethnicity (when applicable).

DNA methylation analysis for biomarker applications is often based on dichotomized variables, which in a clinical setting eases interpretation of test results for individual patients. However, it has been argued that dichotomization of variables should not be used in clinical research (including biomarker discovery studies), as cut-point models invariably lead to loss of information and statistical power, as well as increase the probability of false positive results [90]. Moreover, cut-points such as the “median” or “75th percentile” are inherently data-dependent, and the exact cut-off value will thus vary between sample sets. Furthermore, data-derived “optimal” cut-points can introduce over-optimism (over-fitting) bias into the data [90]. Accordingly, future studies investigating the prognostic utility of DNA methylation biomarkers as dichotomized variables should report how the cut-off point was obtained as well as the exact cut-off value. Moreover, methylation marker candidates should be investigated in multiple independent cohorts with large sample sizes, providing sufficient power for statistical analysis and thus also validating the cut-off point in independent sample sets. Compliance to these measures would ease comparison of results between studies and cohorts, and thus help facilitate the translation of novel prognostic biomarker candidates into actual clinical utility. Furthermore, a well-defined clinical end-point is essential. Notably, the very definition of BCR varied between studies reviewed here. In addition, analyses of more clinically relevant end-points are needed, including metastasis, cancer-specific survival, and overall survival. To allow this, it is crucial that cohorts have sufficient follow-up for such analyses, which due to the slowly progressing nature of PC should be at least 15 years [3]. 

Due to technological advancement, new methods are making it possible to profile epigenetic alterations at a genome-wide scale with high sensitivity, using e.g., microarray technology or NGS-based methods. Whereas previous methods, such as MSP or qMSP, were largely restricted to analysis of a few genes, the new platforms have potentiated global DNA methylome analysis in cancer and normal tissue. Such studies have led to the discovery of genome-wide aberrant DNA methylation patterns in PC, and identification of a large number of DNA methylation changes, many of which have potential as novel diagnostic and some also as prognostic biomarkers [14,74,75,78,91,92,93]. In general, these studies have reported an increase in DNA methylation in PC compared to non-malignant tissue, specifically centered at CGIs and promoter regions, and studies have found an overall inverse correlation between promoter DNA methylation and gene expression [78,91,92]. Moreover, increasing levels of CGI DNA methylation has been found to correlate with increasing disease severity [78,92].

Age is a major risk factor for many cancers, including PC. Interestingly, it has also been established that DNA methylation patterns are subject to age-related alterations, seemingly due to stochastic processes as well as environmental exposures. The aging methylome is characterized by a genome-wide loss of DNA methylation, accompanied by site-specific hypermethylation, similar to the changes observed in cancer. Moreover, most age-related hypermethylation events occur in CGIs, whereas the hypomethylation mainly targets non-CGI regions, and this pattern appears to be consistent across different tissues [94]. Age dependent DNA methylation alters cell physiology and may predispose to malignant transformation [26], and it has been proposed that age induced alterations in DNA methylation may contribute to PC development [95]. Whether age-related changes in methylation can be used as biomarkers for cancer remains to be investigated.

PC often arises in multiple foci from independent precursor cells [96]. The highly heterogeneous and multifocal nature of this disease poses extra challenges to clinicians and researchers, as biopsy samples taken from the prostate may not represent the most clinically relevant lesion and may also not reflect intratumor heterogenetity. Molecular analysis of liquid tumor biopsies (blood, urine or other body fluids) may present a solution to this problem, as circulating cell-free DNA in body fluids may better represent the entire tumor burden, potentially “leaking” tumor DNA into the circulation [97].

In healthy subjects, cfDNA has been found at an average of 30 ng/mL blood [98]. Increased levels of cfDNA are generally associated with pregnancy, as well as disease states such as cancer [99], where the average concentration of cfDNA in blood has been reported at ~180 ng/mL [98]. The exact source and release mechanism of DNA into the circulation is unknown. Most cfDNA is thought to originate from apoptotic and necrotic cells, giving rise to short (70–200 bp) and long (<21 kb) DNA fragments, respectively [97]. Another potential mechanism of cfDNA release is thought to be lysis of intact cancer cells that have intravasated into circulation. Moreover, it has been proposed that cancer cells can actively secrete DNA-nucleoprotein complexes into blood [98]. Most cfDNA seem to be double stranded and present in nucleoprotein or vesicular complexes, presumably protecting the DNA from degradation by plasma nucleases [98,100]. Nevertheless, cfDNA is subject to quick removal from circulation, as studies have shown that fetal cfDNA in maternal blood has a half-life in the range of 4–30 min [101]. The clearance is partly due to plasma nucleases, but cfDNA is also removed by the liver, spleen, and kidneys [101]. In addition to blood, cfDNA isolated from urine is a potential candidate for detection of tumor-specific DNA methylation. A potential problem associated with using urine samples as a source of cfDNA, however, is the inherent variance in urine concentration as urine is not subject to homeostatic regulation. Possibly, this could be overcome by adjusting for creatinine content [102]. Finally, another factor that has been reported to have impact on the yield of cfDNA recovery from urine is the use of prostatic massage or palpation, which has been found to improve sensitivity [100].

As discussed in this review, tumor-specific DNA methylation detected in cfDNA in body fluids has shown potential as prognostic biomarkers for PC. However, this field of research is still at an early stage, and somewhat contradictory results have been reported in different studies [31,32,33], but may at least partly be explained by small sample sizes. Moreover, different sampling and storage protocols for biofluids, as well as variability between methods of DNA extraction and quantification may also cause variations in study results [98], as yield and efficiencies of extracted DNA can differ by orders of magnitude due to different methods [99]. In line with this, several studies have highlighted the need for standardized procedures in order to truly unveil the diagnostic and prognostic biomarker potential of cfDNA and, thus, also tumor-specific cfDNA methylation [97,98,99].

In conclusion, several promising prognostic methylation marker candidates have been identified, the most prominent being *PITX2*, *GABRE~miR-452~miR-224*, *C1orf114*, and the marker panel *AOX1/C1orf114/HAPLN3* which have all been reported as independent predictors of BCR after RP. So far, these biomarker candidates have been examined only in tissue samples from RP specimens, and thus have yet to be investigated in needle biopsy specimens or body fluids. Although these top candidate markers provide statistically significant independent predictive values in multivariate analysis, the relative contributions are rather modest with HRs ranging from 1.38 to 3.27. Thus, it remains to be established whether these candidate biomarkers provide sufficient independent prognostic information to justify methylation analysis in the clinic. However, in a preoperative setting, where only clinical factors are available (*i.e.*, biopsy Gleason score, PSA level at diagnosis, and clinical stage), it is likely that the methylation markers would contribute relatively more independent prognostic information than at post-surgery and thus potentially could be useful in guiding preoperative treatment decisions. Moreover, the possible prognostic value in relation to other and perhaps more clinically relevant endpoints (e.g., overall and cancer-specific survival) also remains to be investigated for these candidate methylation markers. Future studies should aim at stringent study designs, including multivariate analysis on samples from large, well-defined cohorts with equally well-defined clinical end-points. The most reliable prognostic information is expected to be obtained from cohorts with long follow-up, potentiating analysis of end-points such as metastasis, PC specific- or overall survival. For studies investigating methylation as a dichotomized variable, the cut-point defining high and low methylation needs to be clearly stated and validated in multiple cohorts.

## 8. Perspectives

Identification of clinically relevant biomarkers for PC remains a major challenge, especially due to the marked heterogeneity and multifocality of this disease. Thus far, no single genetic or epigenetic lesion has been identified as a hallmark of PC, and as of yet, there is no clearly defined phenotype of aggressive PC [89]. However, evidence suggests that PC progresses along a limited number of genetic pathways that may generate distinct PC subtypes [89]. While single markers may lack sensitivity and specificity required for a prognostic test, an increase in the predictive capability can be achieved using biomarker panels [14,51,53,55,59,61]. While this review has focused on the prognostic utility of DNA methylation markers, in the future, a possible scenario could be the use of multi-parametric marker panels, with inclusion of molecular markers, such as DNA methylation, mutations, gene expression signatures, and copy-number variations, into algorithms of existing clinicopathological parameters, thus potentially generating more robust tools for predicting PC outcome [89]. Such biomarker panels may also be applied to liquid biopsies, thus allowing for longitudinal monitoring of disease progression or treatment response in cancer patients. One of the main obstacles for using liquid biopsies in diagnostic and prognostic applications may be the current lack of standardized laboratory procedures. Nevertheless, liquid biopsies hold great potential for future use in medicine, and improved accuracy may be granted using NGS based methods and digital PCR [100].

Finally, although the biological importance and biomarker potential of aberrant promoter hypermethylation in cancer, including PC, is widely appreciated, the possible role of DNA methylation changes beyond promoter CGIs has been almost completely overlooked [19]. This also means that a huge potential source for methylation marker discovery remains to be explored. Furthermore, to fully understand the role of epigenetic reprogramming in PC, it is crucial to develop maps of the complete DNA methylome in both normal prostatic cells and in PC cells with different malignant potential. Whole-genome bisulfite sequencing (WGBS) now allows studies of the full DNA methylome at single-base resolution [103]. WGBS studies have revealed that methylation patterns are highly dynamic also beyond promoters and that the position of methylation in the transcription unit influences its role in gene control [19,104,105]. Studies of normal and cancer cells have also revealed the existence of thousands of hypomethylated regions [105,106,107,108,109], many of which overlap with enhancers critical for determination of cell identity, function, and regulation of key cancer genes [110,111]. Recent findings suggest that enhancer methylation is a better predictor of cancer gene expression than promoter methylation [112] and most known PC susceptibility loci are located in distal enhancers [113]. Accordingly, deeper understanding of epigenetic reprogramming in PC may not only provide new biomarker candidates, but could also form the basis for development of new therapeutic strategies [114].

## References

[1] Crawford E.D. (2003). Epidemiology of prostate cancer. Urology.

[2] SEER Stat Fact Sheets: Prostate Cancer. http://seer.cancer.gov/statfacts/html/prost.html.

[3] Albertsen P.C., Hanley J.A., Fine J. (2005). 20-year outcomes following conservative management of clinically localized prostate cancer. J. Am. Med. Assoc..

[4] Roehl K.A., Han M., Ramos C.G., Antenor J.A., Catalona W.J. (2004). Cancer progression and survival rates following anatomical radical retropubic prostatectomy in 3478 consecutive patients: Long-term results. J. Urol..

[5] Steineck G., Helgesen F., Adolfsson J., Dickman P.W., Johansson J.E., Norlen B.J., Holmberg L., Scandinavian Prostatic Cancer Group Study N. (2002). Quality of life after radical prostatectomy or watchful waiting. N. Engl. J. Med..

[6] Kattan M.W., Eastham J.A., Wheeler T.M., Maru N., Scardino P.T., Erbersdobler A., Graefen M., Huland H., Koh H., Shariat S. (2003). Counseling men with prostate cancer: A nomogram for predicting the presence of small, moderately differentiated, confined tumors. J. Urol..

[7] Steyerberg E.W., Roobol M.J., Kattan M.W., van der Kwast T.H., de Koning H.J., Schroder F.H. (2007). Prediction of indolent prostate cancer: Validation and updating of a prognostic nomogram. J. Urol..

[8] Ploussard G., Epstein J.I., Montironi R., Carroll P.R., Wirth M., Grimm M.O., Bjartell A.S., Montorsi F., Freedland S.J., Erbersdobler A. (2011). The contemporary concept of significant *versus* insignificant prostate cancer. Eur. Urol..

[9] Shariat S.F., Karakiewicz P.I., Roehrborn C.G., Kattan M.W. (2008). An updated catalog of prostate cancer predictive tools. Cancer.

[10] Cooperberg M.R., Simko J.P., Cowan J.E., Reid J.E., Djalilvand A., Bhatnagar S., Gutin A., Lanchbury J.S., Swanson G.P., Stone S. (2013). Validation of a cell-cycle progression gene panel to improve risk stratification in a contemporary prostatectomy cohort. J. Clin. Oncol..

[11] Salami S.S., Schmidt F., Laxman B., Regan M.M., Rickman D.S., Scherr D., Bueti G., Siddiqui J., Tomlins S.A., Wei J.T. (2013). Combining urinary detection of tmprss2:Erg and pca3 with serum psa to predict diagnosis of prostate cancer. Urol. Oncol..

[12] Sartori D.A., Chan D.W. (2014). Biomarkers in prostate cancer: What’s new?. Curr. Opin. Oncol..

[13] Banez L.L., Sun L., van Leenders G.J., Wheeler T.M., Bangma C.H., Freedland S.J., Ittmann M.M., Lark A.L., Madden J.F., Hartman A. (2010). Multicenter clinical validation of pitx2 methylation as a prostate specific antigen recurrence predictor in patients with post-radical prostatectomy prostate cancer. J. Urol..

[14] Haldrup C., Mundbjerg K., Vestergaard E.M., Lamy P., Wild P., Schulz W.A., Arsov C., Visakorpi T., Borre M., Hoyer S. (2013). DNA methylation signatures for prediction of biochemical recurrence after radical prostatectomy of clinically localized prostate cancer. J. Clin. Oncol..

[15] Kristensen H., Haldrup C., Strand S., Mundbjerg K., Mortensen M.M., Thorsen K., Ostenfeld M.S., Wild P.J., Arsov C., Goering W. (2014). Hypermethylation of the gabre~mir-452~mir-224 promoter in prostate cancer predicts biochemical recurrence after radical prostatectomy. Clin. Cancer Res..

[16] Weiss G., Cottrell S., Distler J., Schatz P., Kristiansen G., Ittmann M., Haefliger C., Lesche R., Hartmann A., Corman J. (2009). DNA methylation of the pitx2 gene promoter region is a strong independent prognostic marker of biochemical recurrence in patients with prostate cancer after radical prostatectomy. J. Urol..

[17] Li E., Bestor T.H., Jaenisch R. (1992). Targeted mutation of the DNA methyltransferase gene results in embryonic lethality. Cell.

[18] Wolffe A.P., Matzke M.A. (1999). Epigenetics: Regulation through repression. Science.

[19] Jones P.A. (2012). Functions of DNA methylation: Islands, start sites, gene bodies and beyond. Nat. Rev..

[20] Baylin S.B., Jones P.A. (2011). A decade of exploring the cancer epigenome—Biological and translational implications. Nat. Rev..

[21] Dobosy J.R., Roberts J.L., Fu V.X., Jarrard D.F. (2007). The expanding role of epigenetics in the development, diagnosis and treatment of prostate cancer and benign prostatic hyperplasia. J. Urol..

[22] Baylin S.B., Esteller M., Rountree M.R., Bachman K.E., Schuebel K., Herman J.G. (2001). Aberrant patterns of DNA methylation, chromatin formation and gene expression in cancer. Hum. Mol. Genet..

[23] Aryee M.J., Liu W., Engelmann J.C., Nuhn P., Gurel M., Haffner M.C., Esopi D., Irizarry R.A., Getzenberg R.H., Nelson W.G. (2013). DNA methylation alterations exhibit intraindividual stability and interindividual heterogeneity in prostate cancer metastases. Sci. Transl. Med..

[24] Nelson W.G., Yegnasubramanian S., Agoston A.T., Bastian P.J., Lee B.H., Nakayama M., De Marzo A.M. (2007). Abnormal DNA methylation, epigenetics, and prostate cancer. Front. Biosci..

[25] Issa J.P. (2012). DNA methylation as a clinical marker in oncology. J. Clin. Oncol..

[26] Li L.C., Carroll P.R., Dahiya R. (2005). Epigenetic changes in prostate cancer: Implication for diagnosis and treatment. J. Nat. Cancer Inst..

[27] Phe V., Cussenot O., Roupret M. (2010). Methylated genes as potential biomarkers in prostate cancer. BJU Int..

[28] Brocks D., Assenov Y., Minner S., Bogatyrova O., Simon R., Koop C., Oakes C., Zucknick M., Lipka D.B., Weischenfeldt J. (2014). Intratumor DNA methylation heterogeneity reflects clonal evolution in aggressive prostate cancer. Cell Rep..

[29] Herman J.G., Graff J.R., Myohanen S., Nelkin B.D., Baylin S.B. (1996). Methylation-specific pcr: A novel PCR assay for methylation status of CpG islands. Proc. Natl. Acad. Sci. USA.

[30] Stewart G.D., van Neste L., Delvenne P., Delree P., Delga A., McNeill S.A., O’Donnell M., Clark J., van Criekinge W., Bigley J. (2013). Clinical utility of an epigenetic assay to detect occult prostate cancer in histopathologically negative biopsies: Results of the matloc study. J. Urol..

[31] Bastian P.J., Palapattu G.S., Lin X., Yegnasubramanian S., Mangold L.A., Trock B., Eisenberger M.A., Partin A.W., Nelson W.G. (2005). Preoperative serum DNA gstp1 CpG island hypermethylation and the risk of early prostate-specific antigen recurrence following radical prostatectomy. Clin. Cancer Res..

[32] Ellinger J., Haan K., Heukamp L.C., Kahl P., Buttner R., Muller S.C., von Ruecker A., Bastian P.J. (2008). CpG island hypermethylation in cell-free serum DNA identifies patients with localized prostate cancer. Prostate.

[33] Okegawa T., Nutahara K., Higashihara E. (2010). Association of circulating tumor cells with tumor-related methylated DNA in patients with hormone-refractory prostate cancer. Int. J. Urol..

[34] Payne S.R., Serth J., Schostak M., Kamradt J., Strauss A., Thelen P., Model F., Day J.K., Liebenberg V., Morotti A. (2009). DNA methylation biomarkers of prostate cancer: Confirmation of candidates and evidence urine is the most sensitive body fluid for non-invasive detection. Prostate.

[35] Roupret M., Hupertan V., Catto J.W., Yates D.R., Rehman I., Proctor L.M., Phillips J., Meuth M., Cussenot O., Hamdy F.C. (2008). Promoter hypermethylation in circulating blood cells identifies prostate cancer progression. Int. J. Cancer.

[36] Goessl C., Krause H., Muller M., Heicappell R., Schrader M., Sachsinger J., Miller K. (2000). Fluorescent methylation-specific polymerase chain reaction for DNA-based detection of prostate cancer in bodily fluids. Cancer Res..

[37] Chiam K., Ricciardelli C., Bianco-Miotto T. (2014). Epigenetic biomarkers in prostate cancer: Current and future uses. Cancer Lett..

[38] Goering W., Kloth M., Schulz W.A. (2012). DNA methylation changes in prostate cancer. Method. Mol. Biol.

[39] Maier S., Nimmrich I., Koenig T., Eppenberger-Castori S., Bohlmann I., Paradiso A., Spyratos F., Thomssen C., Mueller V., Nahrig J. (2007). DNA-methylation of the homeodomain transcription factor pitx2 reliably predicts risk of distant disease recurrence in tamoxifen-treated, node-negative breast cancer patients—Technical and clinical validation in a multi-centre setting in collaboration with the european organisation for research and treatment of cancer (eortc) pathobiology group. Eur. J. Cancer.

[40] Vinarskaja A., Schulz W.A., Ingenwerth M., Hader C., Arsov C. (2013). Association of pitx2 mrna down-regulation in prostate cancer with promoter hypermethylation and poor prognosis. Urol. Oncol..

[41] Schayek H., Bentov I., Jacob-Hirsch J., Yeung C., Khanna C., Helman L.J., Plymate S.R., Werner H. (2012). Global methylation analysis identifies pitx2 as an upstream regulator of the androgen receptor and Igf-I receptor genes in prostate cancer. Horm. Metab. Res..

[42] Anglim P.P., Galler J.S., Koss M.N., Hagen J.A., Turla S., Campan M., Weisenberger D.J., Laird P.W., Siegmund K.D., Laird-Offringa I.A. (2008). Identification of a panel of sensitive and specific DNA methylation markers for squamous cell lung cancer. Mol. Cancer.

[43] Toyota M., Kopecky K.J., Toyota M.O., Jair K.W., Willman C.L., Issa J.P. (2001). Methylation profiling in acute myeloid leukemia. Blood.

[44] Schatz P., Dietrich D., Koenig T., Burger M., Lukas A., Fuhrmann I., Kristiansen G., Stoehr R., Schuster M., Lesche R. (2010). Development of a diagnostic microarray assay to assess the risk of recurrence of prostate cancer based on pitx2 DNA methylation. J. Mol. Diagn..

[45] Dietrich D., Hasinger O., Banez L.L., Sun L., van Leenders G.J., Wheeler T.M., Bangma C.H., Wernert N., Perner S., Freedland S.J. (2013). Development and clinical validation of a real-time pcr assay for pitx2 DNA methylation to predict prostate-specific antigen recurrence in prostate cancer patients following radical prostatectomy. J. Mol. Diagn..

[46] Aoki K., Taketo M.M. (2007). Adenomatous polyposis coli (APC): A multi-functional tumor suppressor gene. J. Cell Sci..

[47] Chen Y., Li J., Yu X., Li S., Zhang X., Mo Z., Hu Y. (2013). APC gene hypermethylation and prostate cancer: A systematic review and meta-analysis. Eur. J. Hum. Genet..

[48] Alumkal J.J., Zhang Z., Humphreys E.B., Bennett C., Mangold L.A., Carducci M.A., Partin A.W., Garrett-Mayer E., DeMarzo A.M., Herman J.G. (2008). Effect of DNA methylation on identification of aggressive prostate cancer. Urology.

[49] Ellinger J., Bastian P.J., Jurgan T., Biermann K., Kahl P., Heukamp L.C., Wernert N., Muller S.C., von Ruecker A. (2008). CpG island hypermethylation at multiple gene sites in diagnosis and prognosis of prostate cancer. Urology.

[50] Henrique R., Ribeiro F.R., Fonseca D., Hoque M.O., Carvalho A.L., Costa V.L., Pinto M., Oliveira J., Teixeira M.R., Sidransky D. (2007). High promoter methylation levels of apc predict poor prognosis in sextant biopsies from prostate cancer patients. Clin. Cancer Res..

[51] Liu L., Kron K.J., Pethe V.V., Demetrashvili N., Nesbitt M.E., Trachtenberg J., Ozcelik H., Fleshner N.E., Briollais L., van der Kwast T.H. (2011). Association of tissue promoter methylation levels of APC, TGFBETA2, HOXD3 and RASSF1A with prostate cancer progression. Int. J. Cancer.

[52] Moritz R., Ellinger J., Nuhn P., Haese A., Muller S.C., Graefen M., Schlomm T., Bastian P.J. (2013). DNA hypermethylation as a predictor of PSA recurrence in patients with low- and intermediate-grade prostate cancer. Anticancer Res..

[53] Richiardi L., Fiano V., Grasso C., Zugna D., Delsedime L., Gillio-Tos A., Merletti F. (2013). Methylation of APC and GSTP1 in non-neoplastic tissue adjacent to prostate tumour and mortality from prostate cancer. PLoS One.

[54] Richiardi L., Fiano V., Vizzini L., de Marco L., Delsedime L., Akre O., Tos A.G., Merletti F. (2009). Promoter methylation in APC, RUNX3, and GSTP1 and mortality in prostate cancer patients. J. Clin. Oncol..

[55] Rosenbaum E., Hoque M.O., Cohen Y., Zahurak M., Eisenberger M.A., Epstein J.I., Partin A.W., Sidransky D. (2005). Promoter hypermethylation as an independent prognostic factor for relapse in patients with prostate cancer following radical prostatectomy. Clin. Cancer Res..

[56] Yegnasubramanian S., Kowalski J., Gonzalgo M.L., Zahurak M., Piantadosi S., Walsh P.C., Bova G.S., de Marzo A.M., Isaacs W.B., Nelson W.G. (2004). Hypermethylation of CpG islands in primary and metastatic human prostate cancer. Cancer Res..

[57] Cottrell S., Jung K., Kristiansen G., Eltze E., Semjonow A., Ittmann M., Hartmann A., Stamey T., Haefliger C., Weiss G. (2007). Discovery and validation of 3 novel DNA methylation markers of prostate cancer prognosis. J. Urol..

[58] Stott-Miller M., Zhao S., Wright J.L., Kolb S., Bibikova M., Klotzle B., Ostrander E.A., Fan J.B., Feng Z., Stanford J.L. (2014). Validation study of genes with hypermethylated promoter regions associated with prostate cancer recurrence. Cancer Epidemiol. Biomark. Prev..

[59] Woodson K., O’Reilly K.J., Ward D.E., Walter J., Hanson J., Walk E.L., Tangrea J.A. (2006). CD44 and PTGS2 methylation are independent prognostic markers for biochemical recurrence among prostate cancer patients with clinically localized disease. Epigenetics.

[60] Kron K.J., Liu L., Pethe V.V., Demetrashvili N., Nesbitt M.E., Trachtenberg J., Ozcelik H., Fleshner N.E., Briollais L., van der Kwast T.H. (2010). DNA methylation of HOXD3 as a marker of prostate cancer progression. Lab. Investig..

[61] Kron K., Liu L., Trudel D., Pethe V., Trachtenberg J., Fleshner N., Bapat B., van der Kwast T. (2012). Correlation of ERG expression and DNA methylation biomarkers with adverse clinicopathologic features of prostate cancer. Clin. Cancer Res..

[62] Rizzo M.T. (2011). Cyclooxygenase-2 in oncogenesis. Clin. Chim. Acta.

[63] Bastian P.J., Ellinger J., Wellmann A., Wernert N., Heukamp L.C., Muller S.C., von Ruecker A. (2005). Diagnostic and prognostic information in prostate cancer with the help of a small set of hypermethylated gene loci. Clin. Cancer Res..

[64] Vasiljevic N., Wu K., Brentnall A.R., Kim D.C., Thorat M.A., Kudahetti S.C., Mao X., Xue L., Yu Y., Shaw G.L. (2011). Absolute quantitation of DNA methylation of 28 candidate genes in prostate cancer using pyrosequencing. Dis. Markers.

[65] Moison C., Assemat F., Daunay A., Tost J., Guieysse-Peugeot A.L., Arimondo P.B. (2014). Synergistic chromatin repression of the tumor suppressor gene rarb in human prostate cancers. Epigenetics.

[66] Hesson L.B., Cooper W.N., Latif F. (2007). The role of rassf1a methylation in cancer. Dis. Markers.

[67] Kron K., Pethe V., Briollais L., Sadikovic B., Ozcelik H., Sunderji A., Venkateswaran V., Pinthus J., Fleshner N., van der Kwast T. (2009). Discovery of novel hypermethylated genes in prostate cancer using genomic cpg island microarrays. PLoS One.

[68] Oncomine Homepage. https://www.oncomine.org/resource/main.html.

[69] Van Neste L., Herman J.G., Otto G., Bigley J.W., Epstein J.I., Van Criekinge W. (2012). The epigenetic promise for prostate cancer diagnosis. Prostate.

[70] Nakayama M., Gonzalgo M.L., Yegnasubramanian S., Lin X., De Marzo A.M., Nelson W.G. (2004). Gstp1 CpG island hypermethylation as a molecular biomarker for prostate cancer. J. Cell. Biochem..

[71] Moura C.M., Pontes J., Reis S.T., Viana N.I., Morais D.R., Dip N., Katz B., Srougi M., Leite K.R. (2014). Expression profile of standard and variants forms of CD44 related to prostate cancer behavior. Int. J. Biol. Markers.

[72] Hulf T., Sibbritt T., Wiklund E.D., Patterson K., Song J.Z., Stirzaker C., Qu W., Nair S., Horvath L.G., Armstrong N.J. (2013). Epigenetic-induced repression of microrna-205 is associated with med1 activation and a poorer prognosis in localized prostate cancer. Oncogene.

[73] Olkhov-Mitsel E., Van der Kwast T., Kron K.J., Ozcelik H., Briollais L., Massey C., Recker F., Kwiatkowski M., Fleshner N.E., Diamandis E.P. (2012). Quantitative DNA methylation analysis of genes coding for kallikrein-related peptidases 6 and 10 as biomarkers for prostate cancer. Epigenetics.

[74] Mahapatra S., Klee E.W., Young C.Y., Sun Z., Jimenez R.E., Klee G.G., Tindall D.J., Donkena K.V. (2012). Global methylation profiling for risk prediction of prostate cancer. Clin. Cancer Res..

[75] Kim J.W., Kim S.T., Turner A.R., Young T., Smith S., Liu W., Lindberg J., Egevad L., Gronberg H., Isaacs W.B. (2012). Identification of new differentially methylated genes that have potential functional consequences in prostate cancer. PLoS One.

[76] Vasiljevic N., Ahmad A.S., Beesley C., Thorat M.A., Fisher G., Berney D.M., Moller H., Yu Y., Lu Y.J., Cuzick J. (2013). Association between DNA methylation of hspb1 and death in low gleason score prostate cancer. Prostate Cancer Prostatic Dis..

[77] Pierconti F., Martini M., Pinto F., Cenci T., Capodimonti S., Calarco A., Bassi P.F., Larocca L.M. (2011). Epigenetic silencing of SOCS3 identifies a subset of prostate cancer with an aggressive behavior. Prostate.

[78] Lin P.C., Giannopoulou E.G., Park K., Mosquera J.M., Sboner A., Tewari A.K., Garraway L.A., Beltran H., Rubin M.A., Elemento O. (2013). Epigenomic alterations in localized and advanced prostate cancer. Neoplasia.

[79] Chan K.C., Jiang P., Chan C.W., Sun K., Wong J., Hui E.P., Chan S.L., Chan W.C., Hui D.S., Ng S.S. (2013). Noninvasive detection of cancer-associated genome-wide hypomethylation and copy number aberrations by plasma DNA bisulfite sequencing. Proc. Natl. Acad. Sci. USA.

[80] Wu T., Giovannucci E., Welge J., Mallick P., Tang W.Y., Ho S.M. (2011). Measurement of gstp1 promoter methylation in body fluids may complement PSA screening: A meta-analysis. Br. J. Cancer.

[81] Prensner J.R., Rubin M.A., Wei J.T., Chinnaiyan A.M. (2012). Beyond PSA: The next generation of prostate cancer biomarkers. Sci. Transl. Med..

[82] Schroder F.H., Hugosson J., Roobol M.J., Tammela T.L., Ciatto S., Nelen V., Kwiatkowski M., Lujan M., Lilja H., Zappa M. (2009). Screening and prostate-cancer mortality in a randomized european study. N. Engl. J. Med..

[83] Vestergaard E.M., Nexo E., Torring N., Borre M., Orntoft T.F., Sorensen K.D. (2010). Promoter hypomethylation and upregulation of trefoil factors in prostate cancer. Int. J. Cancer.

[84] Sorensen K.D., Wild P.J., Mortezavi A., Adolf K., Torring N., Heeboll S., Ulhoi B.P., Ottosen P., Sulser T., Hermanns T. (2009). Genetic and epigenetic SLC18A2 silencing in prostate cancer is an independent adverse predictor of biochemical recurrence after radical prostatectomy. Clin. Cancer Res..

[85] Trock B.J., Brotzman M.J., Mangold L.A., Bigley J.W., Epstein J.I., McLeod D., Klein E.A., Jones J.S., Wang S., McAskill T. (2012). Evaluation of GSTP1 and APC methylation as indicators for repeat biopsy in a high-risk cohort of men with negative initial prostate biopsies. BJU Int..

[86] Truong M., Yang B., Livermore A., Wagner J., Weeratunga P., Huang W., Dhir R., Nelson J., Lin D.W., Jarrard D.F. (2013). Using the epigenetic field defect to detect prostate cancer in biopsy negative patients. J. Urol..

[87] Bastian P.J., Ellinger J., Heukamp L.C., Kahl P., Muller S.C., von Rucker A. (2007). Prognostic value of CpG island hypermethylation at PTGS2, RAR-beta, EDNRB, and other gene loci in patients undergoing radical prostatectomy. Eur. Urol..

[88] Liu J.W., Nagpal J.K., Jeronimo C., Lee J.E., Henrique R., Kim M.S., Ostrow K.L., Yamashita K., van Criekinge V., Wu G. (2008). Hypermethylation of *MCAM* gene is associated with advanced tumor stage in prostate cancer. Prostate.

[89] Donovan M.J., Cordon-Cardo C. (2014). Genomic analysis in active surveillance: Predicting high-risk disease using tissue biomarkers. Curr. Opin. Urol..

[90] Royston P., Altman D.G., Sauerbrei W. (2006). Dichotomizing continuous predictors in multiple regression: A bad idea. Stat. Med..

[91] Borno S.T., Fischer A., Kerick M., Falth M., Laible M., Brase J.C., Kuner R., Dahl A., Grimm C., Sayanjali B. (2012). Genome-wide DNA methylation events in TMPRSS2-ERG fusion-negative prostate cancers implicate an EZH2-dependent mechanism with mir-26a hypermethylation. Cancer Discov..

[92] Kim J.H., Dhanasekaran S.M., Prensner J.R., Cao X., Robinson D., Kalyana-Sundaram S., Huang C., Shankar S., Jing X., Iyer M. (2011). Deep sequencing reveals distinct patterns of DNA methylation in prostate cancer. Genome Res..

[93] Kobayashi Y., Absher D.M., Gulzar Z.G., Young S.R., McKenney J.K., Peehl D.M., Brooks J.D., Myers R.M., Sherlock G. (2011). DNA methylation profiling reveals novel biomarkers and important roles for DNA methyltransferases in prostate cancer. Genome Res..

[94] Rang F.J., Boonstra J. (2014). Causes and consequences of age-related changes in DNA methylation: A role for ROS?. Biology.

[95] Kwabi-Addo B., Chung W., Shen L., Ittmann M., Wheeler T., Jelinek J., Issa J.P. (2007). Age-related DNA methylation changes in normal human prostate tissues. Clin. Cancer Res..

[96] Lindberg J., Klevebring D., Liu W., Neiman M., Xu J., Wiklund P., Wiklund F., Mills I.G., Egevad L., Gronberg H. (2013). Exome sequencing of prostate cancer supports the hypothesis of independent tumour origins. Eur. Urol..

[97] Gonzalez-Masia J.A., Garcia-Olmo D., Garcia-Olmo D.C. (2013). Circulating nucleic acids in plasma and serum (CNAPS): Applications in oncology. Oncol. Targets Ther..

[98] Gormally E., Caboux E., Vineis P., Hainaut P. (2007). Circulating free DNA in plasma or serum as biomarker of carcinogenesis: Practical aspects and biological significance. Mutat. Res..

[99] Devonshire A.S., Whale A.S., Gutteridge A., Jones G., Cowen S., Foy C.A., Huggett J.F. (2014). Towards standardisation of cell-free DNA measurement in plasma: Controls for extraction efficiency, fragment size bias and quantification. Anal. Bioanal. Chem..

[100] Ralla B., Stephan C., Meller S., Dietrich D., Kristiansen G., Jung K. (2014). Nucleic acid-based biomarkers in body fluids of patients with urologic malignancies. Crit. Rev. Clin. Lab. Sci..

[101] Lo Y.M., Zhang J., Leung T.N., Lau T.K., Chang A.M., Hjelm N.M. (1999). Rapid clearance of fetal DNA from maternal plasma. Am. J. Hum. Genet..

[102] Narayanan S., Appleton H.D. (1980). Creatinine: A review. Clin. Chem..

[103] Yu Y.P., Ding Y., Chen R., Liao S.G., Ren B.G., Michalopoulos A., Michalopoulos G., Nelson J., Tseng G.C., Luo J.H. (2013). Whole-genome methylation sequencing reveals distinct impact of differential methylations on gene transcription in prostate cancer. Am. J. Pathol..

[104] Timp W., Feinberg A.P. (2013). Cancer as a dysregulated epigenome allowing cellular growth advantage at the expense of the host. Nat. Rev..

[105] Stadler M.B., Murr R., Burger L., Ivanek R., Lienert F., Scholer A., van Nimwegen E., Wirbelauer C., Oakeley E.J., Gaidatzis D. (2011). DNA-binding factors shape the mouse methylome at distal regulatory regions. Nature.

[106] Ziller M.J., Gu H., Muller F., Donaghey J., Tsai L.T., Kohlbacher O., de Jager P.L., Rosen E.D., Bennett D.A., Bernstein B.E. (2013). Charting a dynamic DNA methylation landscape of the human genome. Nature.

[107] Hon G.C., Rajagopal N., Shen Y., McCleary D.F., Yue F., Dang M.D., Ren B. (2013). Epigenetic memory at embryonic enhancers identified in DNA methylation maps from adult mouse tissues. Nat. Egenet..

[108] Xie W., Schultz M.D., Lister R., Hou Z., Rajagopal N., Ray P., Whitaker J.W., Tian S., Hawkins R.D., Leung D. (2013). Epigenomic analysis of multilineage differentiation of human embryonic stem cells. Cell.

[109] Jeong M., Sun D., Luo M., Huang Y., Challen G.A., Rodriguez B., Zhang X., Chavez L., Wang H., Hannah R. (2014). Large conserved domains of low DNA methylation maintained by dnmt3a. Nat. Genet..

[110] Hnisz D., Abraham B.J., Lee T.I., Lau A., Saint-Andre V., Sigova A.A., Hoke H.A., Young R.A. (2013). Super-enhancers in the control of cell identity and disease. Cell.

[111] Loven J., Hoke H.A., Lin C.Y., Lau A., Orlando D.A., Vakoc C.R., Bradner J.E., Lee T.I., Young R.A. (2013). Selective inhibition of tumor oncogenes by disruption of super-enhancers. Cell.

[112] Aran D., Sabato S., Hellman A. (2013). DNA methylation of distal regulatory sites characterizes dysregulation of cancer genes. Genome Biol..

[113] Aran D., Hellman A. (2014). Unmasking risk loci: DNA methylation illuminates the biology of cancer predisposition: Analyzing DNA methylation of transcriptional enhancers reveals missed regulatory links between cancer risk loci and genes. BioEssays.

[114] Jones P.A. (2014). At the tipping point for epigenetic therapies in cancer. J. Clin. Investig..

